# Supplementary material for: Predicting mental health problems in adolescence using machine learning techniques
Source: PLoS One. 2020 Apr 6;15(4):e0230389. doi: 10.1371/journal.pone.0230389 (PMC7135284; doi:10.1371/journal.pone.0230389)
Supplement: S1 Table — Optimal and explored parameters for the support vector machine model. (DOCX) [file pone.0230389.s002.docx]

**S1 Table**. **Support Vector Machine.**

| **Parameter** | **R function name** | | **value** |
| --- | --- | --- | --- |
| C | | C | 0.1 |
| Gamma | | gamma | 0.01315789 |

C is the penalty of the error term

Gamma can be thought of as the flexibility of the model, with variance increasing as the value gets higher
