# Supplementary material for: Predicting mental health problems in adolescence using machine learning techniques
Source: PLoS One. 2020 Apr 6;15(4):e0230389. doi: 10.1371/journal.pone.0230389 (PMC7135284; doi:10.1371/journal.pone.0230389)
Supplement: S2 Table — Optimal and explored parameters for the neural network model. (DOCX) [file pone.0230389.s003.docx]

**S2 Table**. Neural network.

| **Parameter** | **R function name** | | **value** |
| --- | --- | --- | --- |
| Repetitions | | rep | 6 |
| Hidden Neurons | | hidden | 3 |
| Learning rate | | learningrate | 0.164 |
| Stepmax | | stepmax | 9458.822663 |
| Threshold | | threshold | 0.29 |

Repetition is the number of repetitions in the training process

Hidden neurons in the number of neurons in each layer

Learning Rate is the amount of weights that are updated during training

Stepmax is the maxiumum number of “steps” the model can take, once the model has reached the maximum number of steps it automatically ends the training process.

Threshold specifies the error function for stopping criteria
