# Supplementary material for: Predicting mental health problems in adolescence using machine learning techniques
Source: PLoS One. 2020 Apr 6;15(4):e0230389. doi: 10.1371/journal.pone.0230389 (PMC7135284; doi:10.1371/journal.pone.0230389)
Supplement: S3 Table — Optimal and explored parameters for the random forest model. (DOCX) [file pone.0230389.s004.docx]

**S3 Table**. Random forest.

| **Parameter** | **R function name** | | **value** |
| --- | --- | --- | --- |
| Number of Trees | | ntree | 3485 |
| Node Size | | nodesize | 3 |
| Mtry | | mtry | 14 |

Number of trees refers to the number of decision trees within the random forest model

Node size is the smallest size of the terminal node, thus the larger the parameter the smaller the tree

Mtry is the number of variables the model can split at each node
