# Supplementary material for: Predicting mental health problems in adolescence using machine learning techniques
Source: PLoS One. 2020 Apr 6;15(4):e0230389. doi: 10.1371/journal.pone.0230389 (PMC7135284; doi:10.1371/journal.pone.0230389)
Supplement: S4 Table — Optimal and explored parameters for the XGBoost model. (DOCX) [file pone.0230389.s005.docx]

**S4 Table**. XGBoost

| **Parameter** | **R function name** | | **value** | | |
| --- | --- | --- | --- | --- | --- |
| Maximum Depth | | Max_depth | | | 12 |
| Number of rounds | | nrounds | | | 4 |
| Eta | | eta | | 0.3051818 | |
| Subsample | | subsample | | 0.2488308 | |
| Minimum Child Weight | | Min_child_weight | | 4.745694 | |
| Column sample by tree | | Colsample_bytree | | 0.4381284 | |
| Lambda | | lambda | | 0.5728013 | |

Maximum Depth is the maximum depth of the tree, the bigger the tree the more complex the model

Number of rounds indicates the number of rounds for boosting

Eta shrinks the feature weights to prevent overfitting in the boosting process

Minimum Child Weight limits the partition of future nodes in the tree

Column sample by tree is the ratio of features sampled when building trees

Lambda is the regularization term on weights
